# Evaluation of Food Safety Practices and Microbiological Quality of Street Foods in Marrakech, Morocco

**DOI:** 10.1002/fsn3.70322

**Published:** 2025-06-12

**Authors:** Morad Kaddouri, Omar Ait El Alia, Yassine Zine‐eddine, Ali S. Alqahtani, Mohamed Bouhrim, Aziz Galman, Khalid Boutoial

**Affiliations:** ^1^ Laboratory of the Engineering and Applied Technologies, Higher School of Technology Sultan Moulay Slimane University Beni Mellal Morocco; ^2^ Department of Pharmacognosy, College of Pharmacy King Saud University Riyadh Saudi Arabia; ^3^ Biological Engineering Laboratory, Faculty of Sciences and Techniques Sultan Moulay Slimane University Beni Mellal Morocco; ^4^ Laboratoires TBC, Laboratory of Pharmacology, Pharmacokinetics, and Clinical Pharmacy, Faculty of Pharmaceutical and Biological Sciences University of Lille Lille France

**Keywords:** food safety practices, foodborne pathogens, hygiene standards, Marrakech, microbiological quality, street foods

## Abstract

This study investigates food safety practices and evaluates the microbiological quality of street foods marketed in Marrakech, Morocco. Data collection involved an observational checklist to systematically assess hygiene practices, environmental conditions, and the operational characteristics of street food vendors. Furthermore, microbiological analyses were conducted on 224 ready‐to‐eat food samples to determine their compliance with Moroccan food safety standards. The results revealed that 21% of the samples were non‐compliant with these standards, with *Escherichia coli* (28%) and fecal coliforms (40%) identified as the predominant contaminants. Statistical analysis using chi‐squared tests demonstrated significant associations between improved food safety practices—such as the use of gloves, hairnets, and appropriate utensil sanitation—and lower levels of microbial contamination. Additionally, environmental factors, particularly the cleanliness of vending sites, exhibited a strong correlation with elevated microbial loads. These findings highlight the pivotal role of hygienic practices and environmental conditions in mitigating foodborne risks. The study underscores the necessity for implementing robust food safety measures and reinforcing regulatory oversight within the street food sector to ensure public health protection.

## Graphical abstract

This study evaluates hygiene practices and microbiological quality of street foods in Marrakech. Among 224 samples analyzed, 21% were non‐compliant with Moroccan safety standards, with contamination dominated by fecal coliforms (40%) and *Escherichia coli* (28%). Statistical analyses demonstrate that glove use, hairnets, and environmental cleanliness significantly reduce microbiological risks.

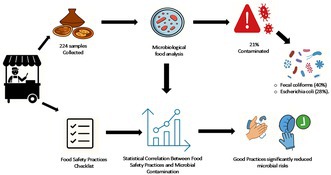

## 1 Introduction

Street food encompasses a variety of foods and beverages prepared and/or sold by vendors in public areas, such as streets, and intended for immediate consumption or for later use without requiring further preparation (FAO 2013; Sgroi et al. 2022). In many developing nations, street food has become a vital aspect of urban life, influenced by the dynamics of urbanization and socio‐economic transformations (Bouafou et al. 2021). This sector significantly contributes to meeting the nutritional needs of urban populations by providing food that is both accessible and often economically viable (Steyn et al. 2014).

In Morocco, the variety and prevalence of street food have expanded, particularly in industrialized regions and cities popular with tourists (Lee et al. 2019; Salamandane et al. 2023). This growth is attributed to factors such as time constraints faced by diverse groups, including tourists, students, workers, the unemployed, street children, and shopkeepers, which make dining outside the home a practical solution (Chatibura 2021). Nevertheless, the rapid proliferation of street food vendors has heightened concerns regarding food safety. Inadequate hygiene practices during food preparation and sales have been linked to an increased risk of microbial contamination and toxin exposure (Compaore et al. 2022).

The safety of street food is determined by several critical factors, including the quality of raw ingredients, compliance with hygiene standards, and adherence to safe preparation practices (Akhter and Cameron 2023). Unfortunately, these standards are often inadequately implemented, resulting in street foods frequently serving as vectors for foodborne illnesses and outbreaks. These outbreaks commonly lead to gastrointestinal diseases such as gastroenteritis and diarrhea, primarily caused by microbial contamination (Wu et al. 2024).

Studies consistently highlight the vulnerability of street foods to environmental hazards such as insects, flies, and air pollution (Alimi 2016). Moreover, many street vendors lack sufficient knowledge of food hygiene practices and often store or display food under suboptimal conditions, increasing the risk of cross‐contamination and compromising food preservation (Cortese et al. 2016). The consumption of contaminated food, tainted with bacteria, viruses, parasites, or toxic chemicals, represents a significant global public health concern. It is linked to over 200 diseases, ranging from diarrhea to cancer (Kagaruki et al. 2021; Nonato et al. 2016). Globally, an estimated 600 million people—approximately 1 in 10—fall ill annually due to the consumption of unsafe food, resulting in 420,000 deaths. Among these fatalities, 125,000 are children under the age of five (WHO 2024).

In Morocco, foodborne illnesses have become an increasingly pressing issue, exacerbated by their rising incidence and growing public awareness (Bencheikh 2009). According to the Epidemic Diseases Department of the Directorate for Disease Control and Epidemiology, reported cases of foodborne illnesses increased steadily from 2008 to 2017, with approximately 1600 cases documented annually (Amaiach et al. 2023). However, this figure likely underrepresents the true burden, as many cases go unreported, with estimates suggesting 10 unreported cases for every documented instance. The most frequently identified pathogens include *Salmonella*, *Staphylococcus* spp., and fecal coliforms (Murgia et al. 2015; Nacer et al. 2024).

Recent reports of foodborne illnesses linked to street foods in Morocco, particularly in Marrakech (Moroccan Ministry of Health 2021), underscore the urgent need to evaluate the microbiological safety of these widely consumed food items by both locals and tourists. This study seeks to systematically assess the hygiene practices and safety measures employed by street food vendors while conducting microbiological analyses of street food products to identify potential contamination risks. Notably, this is the first investigation of its kind focused on the street food sector in Marrakech, Morocco.

## 2 Materials and Methods

### 2.1 Location of the Study

**FIGURE 1:**
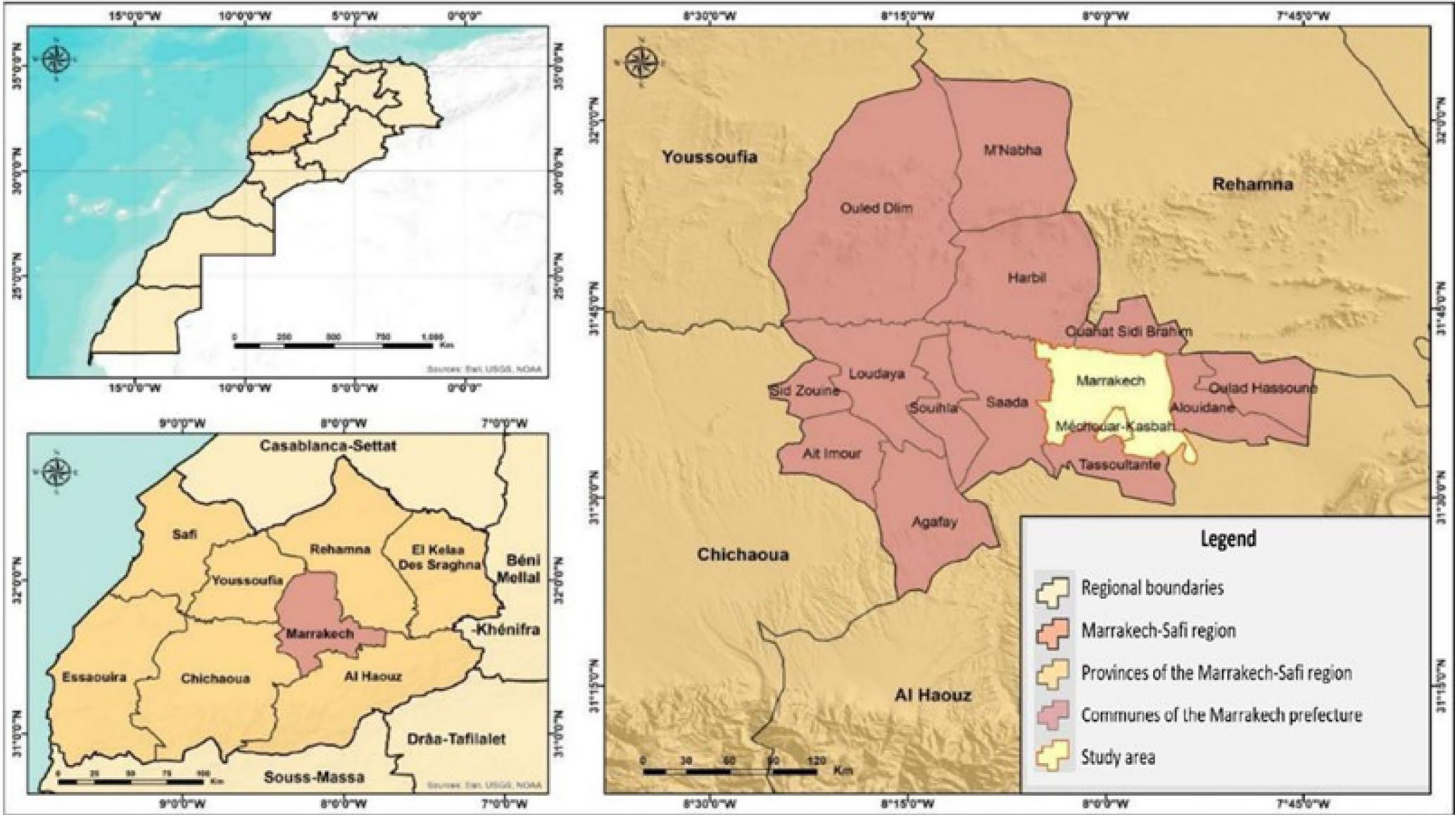
Geographic location of the study area (Marrakech City). *Source:* Satellite map of Marrakech. GPS coordinates: 31°37′43.2264′′ N, 7°59′31.3692′′ W (Rafik 2023).

Marrakech City (Figure 1) is located within the Marrakech‐Safi region and is one of the four largest cities in Morocco. It covers an area of 216 km^2^ and has a population of 1,571,580 inhabitants, according to the 2024 Population and Housing Census of Morocco (Maaroufi 2024). The city is subdivided into five districts: Marrakech‐Medina, Menara, Annakhil, Sidi Youssef Ben Ali, and Guéliz. It is bordered to the north by the communes of Ouahat Sidi Ibrahim and Harbil, to the south by the communes of Méchouar Kasbah and Tasseltante, to the east by the communes of Alouidane and Ouled Hassoun, and to the west by the commune of Saada (Ouhedda 2015; Rafik 2023).

### 2.2 Sampling

A total of 224 ready‐to‐eat street food samples were collected from five major communes within Marrakech: Ennakhil, Guéliz, Marrakech‐Medina, Ménara, and Sidi Youssef Ben Ali. This sampling strategy ensured comprehensive geographical representation, capturing socio‐economic variations across diverse neighborhoods. The collection period extended from January to November 2024.

Sample collection adhered to the General Sampling Guidelines specified in the Moroccan Standard Code NM 08.0.014 (2013), which provides standardized protocols for sampling procedures. Each sample was placed in sterile plastic bags and transported to the laboratory under controlled refrigeration conditions, with temperatures maintained between 1°C and 8°C to prevent microbial proliferation during transit. Upon arrival at the laboratory, the samples were stored in a refrigerator at temperatures ranging from 1°C to 5°C to ensure optimal preservation. Microbiological analyses were conducted within 24 h of collection to preserve the samples' original microbial integrity and minimize the risk of contamination or degradation.

### 2.3 Vendor Classification and Sales Environments

To capture the variability in food handling practices, vendors were classified into three categories according to their sales model and operational settings: (i) Itinerant vendors: These vendors prepare food at home and sell it in various locations without a fixed point of sale. Their operations are highly mobile, and they often rely on informal networks or regular routes within neighborhoods to sell their products; (ii) Semi‐fixed vendors: These vendors prepare and sell food on‐site in open‐air settings such as streets, squares, or local markets. They usually operate with makeshift equipment, and their locations are temporary, which makes food preservation and hygienic practices more challenging; (iii) Fixed vendors: This group includes vendors with permanent establishments such as snack bars, street cafes, or hotel‐based food outlets. They generally have access to better facilities for food preparation, preservation, and hygiene but are still subject to scrutiny regarding cleanliness and compliance with health regulations.

### 2.4 Food Safety Observational Checklist

During sample collection, special attention was given to the vendors' immediate environment and hygiene practices. Observations were made regarding the presence of solid or liquid waste near water sources, any unpleasant odors, and the condition of utensils and dishwater. A checklist was utilized to assess the food safety practices of street food vendors and the environmental conditions of the corresponding vending sites. This checklist, presented in Table 1, provided a systematic overview of hygiene practices and environmental factors that could influence food safety.

**TABLE 1:** Food safety checklists for street foods.

General information
Sampling date
Restaurant name and address
Type of vending site
Itinerant vendors
Semi‐fixed vendors
Fixed vendors
Environment around the vending site
Is environment around the stall clean: far from rubbish, waste water, toilet facilities, open drains and pests?
Yes
No
Personal hygiene
Yes
No
Vendors clothes clean and presentable
Yes
No
Vendors handle food using gloves
Yes
No
Vendors use hair net
Yes
No
Food storage and handling
Food stored/displayed in covered clean containers
Yes
No
Food prepared and served straight away
Yes
No
Raw, partially cooked and cooked food products kept separate
Yes
No
Vendor use the same utensil for all food types
Yes
No
Cleaning and dishwashing
Utensils cleaned properly after use
Yes
No
Sanitizers used for cleaning at least food contact surfaces
Yes
No
Availability and cleanliness of dishwater
Yes
No
Utensils cleaned with warm soapy water
Yes
No
Specific comments
Presence of insects or rodents
Yes
No
Comments

The use of this tool also helped identify specific practices that could increase the risk of contamination, thus facilitating the evaluation of vendors' compliance with basic hygiene standards and the necessary measures to mitigate public health risks.

Based on our experience and a previous study by Wu et al. (2024), the selected elements were deemed most relevant for evaluating the hygienic practices of the ready‐to‐eat street food vendors examined in this study. We prioritized factors that directly impact food safety and hygiene, ensuring a comprehensive assessment that accurately reflects the actual conditions under which these vendors operate. This approach allowed us to gather meaningful data that can inform improvements in food safety practices within this sector.

### 2.5 Classification and Characterization of Samples

The sampled products include meat, fish, fruits and vegetables, dairy items, salads, pastries, egg‐based products, and milk‐based ice cream. The samples were classified following the guidelines (General Secretariat of the Government 2004). This Moroccan regulation specifies the microbiological criteria for various food types and identifies the microorganisms to be evaluated in determining microbiological quality. The samples were categorized (Table 2) to facilitate interpretation. A compliance assessment by food category allowed for the identification of products vulnerable to microbial contamination, underscoring the need for strict hygiene practices during handling.

**TABLE 2:** Classification of samples into groups and the number of samples for each type.

Type of food	Number of samples
Milk and dairy products	19
Pastries and pastry creams	41
Prepared dishes (animal‐based)	45
Raw meats and meat products	17
Semi‐preserved foods	6
Prepared dishes (plant‐based)	40
Seasoning sauces	9
Vegetables and fresh produce	47

### 2.6 Microbiological Analyses

The analysis was conducted in accordance with Moroccan standards for microbiological food analysis (General Secretariat of the Government 2004), involving specific enumeration procedures for each microbial parameter based on the sample type.

The preparation of stock solutions and decimal dilutions was carried out in accordance with the Moroccan standard code NM 6887‐1 (2006). A 25 g sample of each product was weighed using a calibrated balance (uncertainty ±0.001 g), placed in a sterile bag, and mixed with 225 mL of buffered peptone water (BPW). Homogenization was performed for 1 min, followed by serial dilutions (10^−1^ to 10^−4^) in BPW.

Total aerobic mesophilic flora (TAMF) counts were performed on plate count agar (PCA), which was preheated to 47°C, cooled to 37°C, and incubated at 30°C, following NM 08.0.121 (2006). For total and fecal coliforms, samples were inoculated on lactose deoxycholate agar (LDA). Plates were incubated at 37°C for total coliforms and at 44°C for fecal coliforms for 24 h, in accordance with NM 08.0.124 (2012).

For sulfite‐reducing anaerobes (ASR), sulfite polymyxin sulfadiazine agar (SPS) was used, with plates incubated at 46°C for 24 h, following NM ISO 15213‐1. For *Staphylococcus aureus* enumeration, Baird‐Parker Agar was used, with plates incubated at 37°C for 48 h, in accordance with NM 08.0.104 (2021). Yeasts and molds were inoculated on sabouraud agar with gentamicin and incubated at 37°C for 72 h, as per NM 08.0.123 (2006).

For *Salmonella* detection (NM ISO 6579‐1), the procedure was as follows: (i) Pre‐enrichment: Samples were incubated in a non‐selective medium for 24 h at 37°C. (ii) Selective enrichment: 0.1 mL of the pre‐enriched solution was added to Rappaport Vassiliadis (RV) broth and incubated at 42°C for 18–24 h. (iii) Isolation: A drop from the RV broth was streaked onto Hektoen Agar and incubated at 37°C for 18–24 h.

For *Listeria monocytogenes* detection (NM 08.0.110, 2006), the procedure was as follows: (i) Primary enrichment: A 25 g sample was mixed with Fraser II broth and additives, and incubated at 30°C for 24 ± 2 h. (ii) Secondary enrichment: 0.1 mL was transferred to Fraser I broth and incubated at 37°C for 48 ± 2 h. (iii) Isolation and confirmation: Cultures were inoculated on Palcam, Oxford, and Compas Listeria agar plates, incubated at 37°C. Colonies were confirmed through oxidase, catalase, Gram stain, motility tests, β‐hemolysis on blood agar, and further confirmation using the Api Listeria biochemical gallery.

The microbiological analysis results obtained were interpreted according to Moroccan regulatory standards (General Secretariat of the Government 2004); all microbial counts were expressed in colony‐forming units per gram (CFU/g), except for Salmonella and *Listeria monocytogenes* , which were interpreted based on their presence or absence.

### 2.7 Data Analysis

The data collected from observations during sample collection and subsequent microbiological analyses were evaluated using a range of statistical techniques. The chi‐squared test was employed to identify associations between categorical variables, while correspondence analysis was conducted to explore relationships within the dataset, utilizing IBM SPSS Statistics version 25. Additionally, multivariate statistical methods were applied to assess differences across groups. Specifically, a one‐way multivariate analysis of variance was performed, followed by Tukey's test to determine significant group differences at a 0.05 significance level. These analyses were carried out using Minitab software version 18 (Minitab Inc., USA).

## 3 Results

### 3.1 Food Safety Practices in Marrakech Street Foods

The observational checklist revealed significant findings concerning the general food safety conditions of street foods served in Marrakech. This assessment, conducted for the first time in Morocco, included an evaluation of vending site types, hygiene practices, and the adequacy of facilities and their surrounding environments.

The majority of vendors (73%) operated fixed establishments, while 23% were semi‐fixed, and 4% used mobile carts. Regarding environmental conditions, 61% of the establishments were located in clean areas, while 39% were maintained at an average level of cleanliness. In terms of personal hygiene, most vendors exhibited unsatisfactory practices, with 72% preparing and serving food without wearing gloves. Additionally, 60% of the vendors wore clean and presentable clothing, hairnets, and aprons. Furthermore, 56% of the establishments failed to properly separate raw ingredients (uncooked products) from cooked foods.

Overall, 30% of vendors prepared and served food immediately, while 70% prepared food in advance. Moreover, 80% used the same utensils for all types of food, and 70% took measures to cover and protect utensils from contamination. In terms of sanitation, 66% of establishments had an adequate supply of potable water for cleaning utensils, but 65% used dirty aprons or cloths to clean surfaces. Only 30% of vendors washed their utensils with warm soapy water.

### 3.2 Bacteriological Analysis

In this study, microbiological analyses were conducted on 224 samples of ready‐to‐eat food purchased from vendors in various streets of Marrakech. The interpretation of the microbiological results was based on the guidelines of the current Moroccan regulations.

**FIGURE 2:**
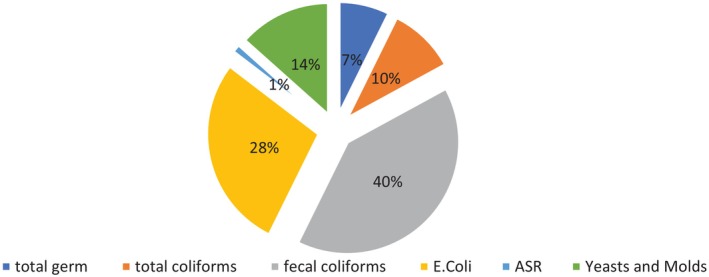
Non‐conformity according to the germs.

Out of the 224 samples tested, 178 (79%) were compliant with Moroccan regulations, while 46 (21%) were non‐compliant. The microorganisms responsible for this non‐compliance are shown in Figure 2, and their distribution was as follows: fecal coliforms (40%), *Escherichia coli* (28%), yeasts and molds (14%), total coliforms (10%), total germs (7%), and ASR (1%). Regarding *Salmonella*, it was detected in only one sample, while no traces of *Listeria* or *S. aureus* were found in any of the tested samples.

**FIGURE 3:**
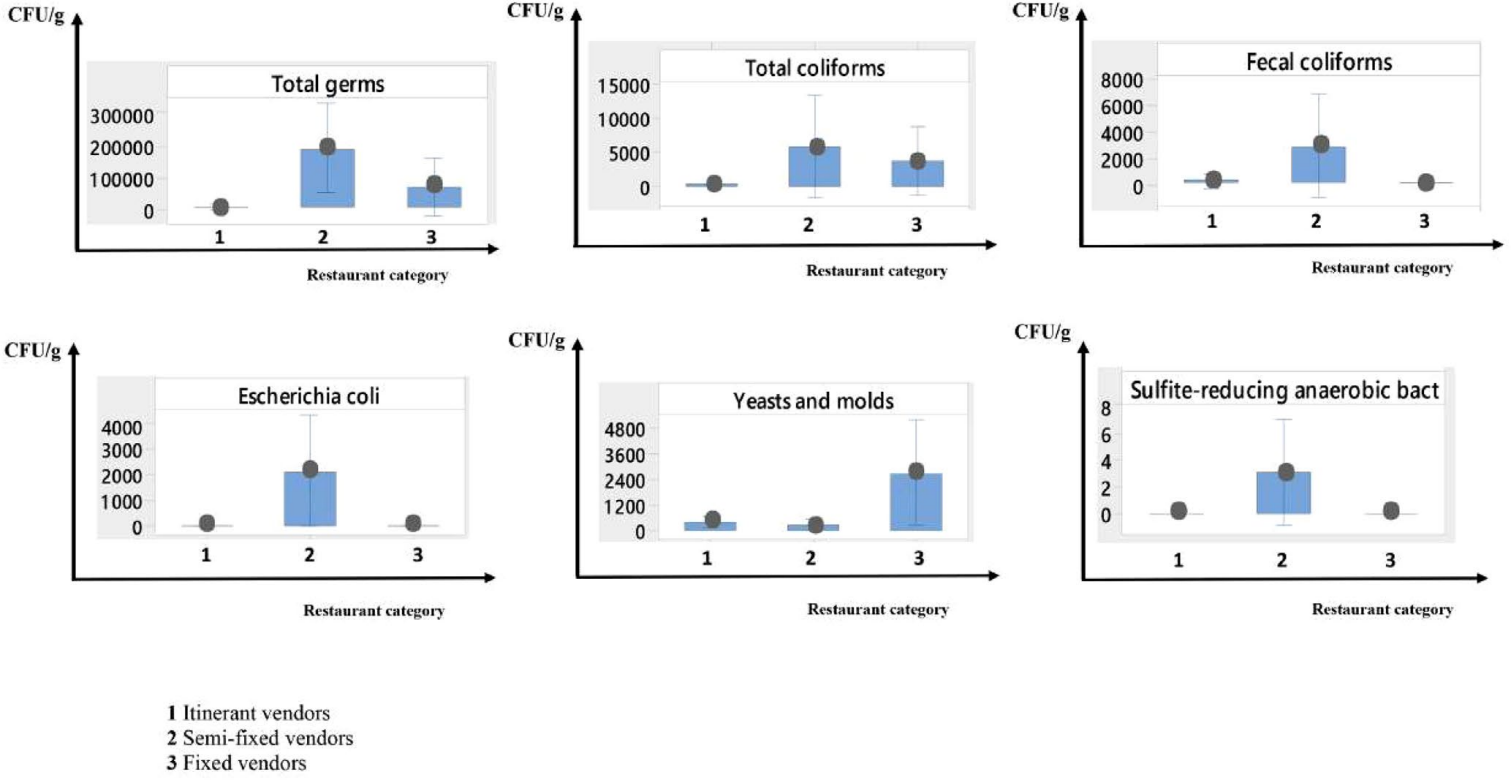
Interval plot of microbiological tests by restaurant category (95% CL for the mean).

Figure 3 illustrates the impact of restaurant type on the microbial load of each microorganism. We observed that all microorganisms were present in significant quantities in fixed restaurants, with total germs exceeding 10^5^ CFU/g, total coliforms surpassing 5 × 10^3^ CFU/g, fecal coliforms exceeding 2 × 10^2^ CFU/g, *E. coli* exceeding 2 × 10^3^ CFU/g, and ASR at 20 CFU/g. In contrast, yeasts and molds (> 2 × 10^3^ CFU/g) were particularly abundant among semi‐fixed vendors, followed by total coliforms at 2 × 10^3^ CFU/g. For mobile vendors, the presence of germs was statistically insignificant compared to the other two restaurant types.

**FIGURE 4:**
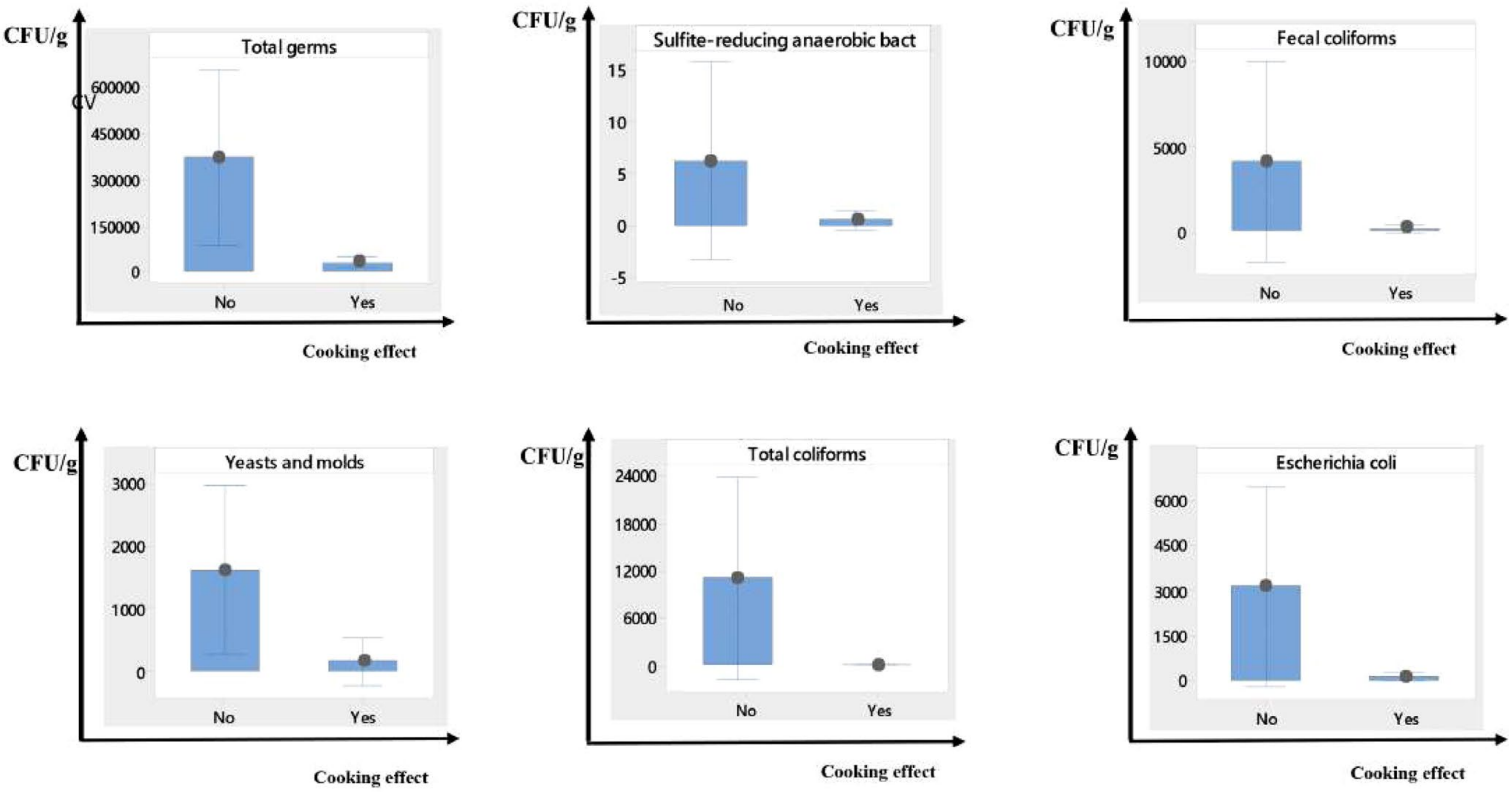
Interval plot of microbiological tests by cooking effect (95% CL for the mean).

Figure 4 shows that raw foods, which have not undergone thermal treatment (cooking), are heavily loaded with the various microorganisms investigated. In contrast, cooked foods exhibit a lower microbial load for all types of microorganisms analyzed.

### 3.3 Correlation Between Food Safety Practices and the Microbiological Quality in Marrakech Street Foods

Chi‐squared tests were applied to evaluate the relationships between the parameters from the food safety observation checklist at each street food vendor and their impact on microbiological analysis outcomes (including total germs, total coliforms, fecal coliforms, *E. coli* , ASR, and product compliance). This analysis revealed a set of dependent variables. Out of 128 chi‐squared tests conducted, 23 were significant dependent variables; all results are summarized in Table 3.

**TABLE 3:** Chi‐squared tests for all parameters of food safety practices, microbiological tests, and product compliance.

	Type of vending site	Nature of sample	Cooking effect	Environmental conditions	Vendors clothes clean and presentable	Vendors use hair net	Vendors handle food using gloves	Food stored/displayed in covered clean containers	Raw, partially cooked and cooked food products kept separate	Vendor use the same utensil for all food types	Food prepared and served straight away	Utensils cleaned properly after use	Utensils cleaned with warm soapy water	Sanitizers used for cleaning at least food contact surfaces	Availability and cleanliness of dishwater	Presence of insects or rodents
Total germ	0.191^a^	553.552^a^	91.225^a^	16.313^a^	26.632^a^	14.580^a^	16.313^a^	3.454^a^	0.064^a^	0.064^a^	0.717^a^	0.072^a^	0.036^a^	0.064^a^	0.064^a^	0.163
Total coliforms	0.083^a^	147.019^a^	39.125^a^	16.432^a^	15.164^a^	14.516^a^	16.432^a^	7.925^a^	0.940^a^	0.940^a^	2.009^a^	0.122^a^	0.122^a^	0.940^a^	0.940^a^	1.488
Fecal coliforms	3.099^a^	114.839^a^	40.866^a^	53.871^b^	74.427^a^	49.113^b^	68.415^b^	9.673^b^	1.465^a^	1.465^a^	8.647^b^	4.708^a^	0.191^a^	1.465^a^	1.465^a^	0.456
*E. coli*	5.564^a^	62.971^a^	23.844^a^	38.522^b^	69.122^a^	35.107^b^	49.053^b^	12.350^b^	0.457^a^	0.457^a^	5.767^a^	3.133^a^	0.127^a^	0.457^a^	0.457^a^	0.046
ASR	0.190^a^	6.483^a^	8.089^a^	2.701^a^	6.994^a^	2.371^a^	2.701^a^	3.744^a^	5.903^a^	5.903^a^	0.128^a^	0.014^a^	0.007^a^	5.903^a^	5.903^a^	6.994
Yeasts and molds	11.500^a^	82.075^a^	9.771^a^	7.857^a^	3.056^a^	7.257^a^	15.337^b^	10.645^a^	9.167^a^	9.167^a^	11.458^b^	17.679^a^	—	9.167^a^	9.167^a^	13.259^b^
*Salmonella*	0.376^a^	17.746^a^	0.902^a^	1.552^a^	5.613^a^	1.441^a^	1.880^a^	2.210^a^	0.384^a^	0.384^a^	0.281^a^	0.109^a^	0.009^a^	0.384^a^	0.384^a^	0.311
Street foods compliance	1.66^a^	16.973^a^	38.655^b^	89.463^b^	85.147^b^	83.025^b^	108.354^b^	39.555^b^	5.369^b^	5.369^b^	0.141^a^	12.979^a^	0.522^a^	5.369^b^	5.369^b^	9.977^b^

^a^ Dependent variables.

^b^ Independent variables.

The contingency coefficient was applied to evaluate the significance of dependent variables, providing an estimate of the degree of association and dependency between two parameters. Among the 23 tests conducted, 11 showed notable contingency values. The findings highlight critical relationships between hygiene practices, environmental conditions, and microbiological quality. Overall environmental cleanliness is strongly associated with the presence of fecal coliforms (coefficient of contingency = 0.461), *E. coli* (coefficient of contingency = 0.405), and general product compliance (coefficient of contingency = 0.534), underscoring the importance of maintaining a sanitary environment. The use of gloves during handling correlates with levels of fecal coliforms (coefficient of contingency = 0.505), *E. coli* (coefficient of contingency = 0.447), yeasts and molds (coefficient of contingency = 0.467), and compliance with product standards (coefficient of contingency = 0.571), suggesting their role in mitigating microbial contamination. Similarly, the wearing of hairnets is linked to reduced total coliform levels (coefficient of contingency = 0.444) and improved product compliance (coefficient of contingency = 0.520), emphasizing their contribution to hygiene practices. Furthermore, the cleaning of utensils before use, along with effective control of pests such as insects and rodents (coefficient of contingency = 0.415), significantly impacts the presence of yeasts and molds (coefficient of contingency = 0.441), highlighting the necessity of thorough sanitation and pest management to ensure food safety and quality.

For the remaining 12 tests, the contingency coefficient values tend toward 0, indicating a weak association between these variables. This suggests that other external factors, not considered in this analysis, may influence the evaluated variables. In future studies, it would be worthwhile to explore these additional factors, such as storage duration or specific preparation methods, to gain a more comprehensive understanding of the determinants of food microbiological quality.

## 4 Discussion

### 4.1 The Current Status of Street Food Operations and the Hygiene Practices Implemented in Marrakech

In this study, the majority of vendors (73%) were observed to operate fixed restaurants, while 23% utilized semi‐fixed setups, and 4% relied on mobile carts. These findings align with previous research, which also identified a predominance of fixed establishments (Loukieh et al. 2018; Wu et al. 2024). However, they contrast with a study conducted in Ethiopia, where semi‐fixed restaurants accounted for the largest proportion (43.5%), followed by fixed establishments (27.4%) and mobile carts (9.7%) (Gemeda et al. 2023). These variations in restaurant types may also be attributed to different regulatory environments and health inspection systems in place across regions. In Morocco, for instance, regular inspections by health services play a critical role in food safety practices, with 20%–25% of food establishments in the catering and retail sectors being at risk each year, as highlighted by the national epidemiological surveillance system (Moroccan Ministry of Health 2021). The Ministry of Health, through its national food hygiene program, has made significant efforts to strengthen foodborne disease monitoring, risk assessment, and awareness campaigns (Rachidi 2020). These efforts may contribute to the predominance of fixed establishments, as they are often subject to stricter regulations compared to mobile vendors, which are more difficult to monitor and control (Rachidi 2011). Furthermore, the infrastructure in Morocco may favor fixed establishments, providing more stability and easier compliance with food safety guidelines (Mouline and Lazrak 2005). These regional factors, including health regulations and consumer expectations, could explain the observed differences in vendor types compared to other regions, such as Ethiopia, where semi‐fixed establishments are more prevalent.

Regarding the environment surrounding the vending sites, 39% of street food vendors do not adhere to the Moroccan standard code: NM 08.0.002, which is the General Guidelines for the HACCP management system. This standard mandates that food vendors must operate in areas that are free from unpleasant odors, smoke, dust, and are situated away from liquid and solid waste or other potential sources of contamination.

The high percentage of vendors (72%) handling food with bare hands observed in this study can be attributed to several factors, including limited access to adequate hygiene facilities, a lack of training or awareness regarding food safety, and the informal nature of street food vending. In many low‐resource settings, vendors often do not have access to running water, hand sanitizers, or gloves, all of which are essential for maintaining hygiene during food handling. Additionally, cultural practices, economic constraints, and weak regulatory enforcement may contribute to the widespread use of bare hands in food preparation. Similar behaviors have been documented in other regions, such as Jigjiga City, Ethiopia (47.62%) (Bereda et al. 2016), and Beirut, Lebanon (86.7%) (Loukieh et al. 2018), where vendors face comparable challenges related to hygiene standards and food safety education. Furthermore, the finding that 40% of vendors in this study did not wear appropriate or clean clothing further highlights the overall lack of hygiene practices in this sector, a trend observed in other countries, including Ethiopia (Adane et al. 2018), Kenya (Muinde and Kuria 2005), and Lebanon (Loukieh et al. 2018). These results underscore the need for improved hygiene infrastructure, targeted awareness campaigns, and more stringent regulatory oversight to mitigate foodborne risks and enhance food safety practices in street food vending.

Good food handling practices are lacking in many street food establishments. Specifically, only 20% of vendors use separate utensils for different types of food, while 56% of restaurants fail to properly separate raw ingredients from cooked foods. Additionally, 30% do not cover or protect utensils from contaminants, 70% prepare food in advance of service, 34% lack a sufficient supply of potable water for cleaning, and only 30% of vendors wash their utensils with warm, soapy water. Furthermore, 65% use dirty aprons or cloths to clean surfaces. These unsafe practices significantly increase the risk of cross‐contamination (Ebert 2018). Effective food segregation and proper utensil hygiene are critical for minimizing contamination risks. Although these practices reduce the chances of contamination and foodborne illness, they are just part of a larger process that, when followed thoroughly at each step, ensures a safe final product (Keely Boyle et al. 2018).

### 4.2 Microbiological Quality of Streets Foods in Marrakech

Microbiological analyses indicate that 21% of the samples tested in our study do not comply with Moroccan standards. This result is comparable to data from a study conducted in the city of Fez between 2003 and 2006, which reported a non‐compliance rate of 34.9% (Marnissi and Bennani 2012). A subsequent study conducted between 2015 and 2019 in the same city observed a reduction in this rate to 31% (Amaiach et al. 2023). In contrast, a much higher non‐compliance rate (67.7%) was reported in a study conducted in Northwest Morocco between 2012 and 2017 (Ghailani et al. 2020). This disparity may suggest significant regional variations in food safety practices, indicating that certain areas face greater challenges in adhering to established food safety standards. These regional differences may be attributed to variations in infrastructure, regulatory enforcement, and local food handling practices. Consequently, there is a clear need for region‐specific interventions and a stronger emphasis on improving food safety education and practices to ensure better public health protection across the country.

The primary bacteria identified were fecal coliforms (40%) and *E. coli* (28%). These findings are consistent with previous studies showing high contamination levels in street foods (Rakha et al. 2022), particularly by bacteria indicative of fecal contamination, underscoring the urgent need to improve food handling and storage practices in street food settings (Barreira et al. 2024). Although the low presence of *Salmonella* and absence of *Listeria* are reassuring, the high levels of *E. coli* and fecal coliforms remain concerning, especially given the food handling and storage conditions observed in this study. The data suggest that inadequate hygiene practices, such as insufficient hand and utensil washing, could be contributing factors to these contaminations.

Additionally, the higher microbial load found in raw foods, compared to cooked foods, confirms that cooking effectively reduces microorganisms. However, the advance preparation of foods before service (practiced by 70% of vendors) exposes the food to an increased risk of recontamination. This finding is supported by studies conducted in developing countries (Rakha et al. 2022), where it has been observed that street foods are often prepared several hours before consumption.

### 4.3 Correlation Between Food Safety Practices and Microbial Contamination of Street Foods in Marrakech

Chi‐squared analyses revealed significant relationships between certain environmental conditions, hygiene practices, and the presence of specific microorganisms. A notable example is the correlation between the sales environment and contamination by fecal coliforms and *E. coli* , which was particularly marked. In our sample, 39% of establishments were located in environments with medium cleanliness, and these establishments showed higher levels of contamination, which is consistent with other similar studies. For example Al Mamun et al. (2013) observed significant levels of contamination by *E. coli* , *Salmonella*, and *Shigella* in foods sold in street environments characterized by poor hygiene conditions, such as insects, dust, or non‐potable water.

The association between hygiene practices and microbiological contamination levels is also evident. The use of gloves, hairnets, and the cleaning of utensils with hot water are strongly correlated with contamination by *E. coli* and total coliforms. Previous studies conducted in Brazil (Ferrari et al. 2021), in Lebanon (Loukieh et al. 2018), in India (Sabbithi et al. 2014), in Bangladesh (Al Mamun et al. 2013), in China (Liu et al. 2014), in Brazil (Magalhães et al. 2017), and in Turkey (Tomar and Akarca 2019) have also demonstrated that adopting proper hygiene practices by food handlers is crucial in preventing food contamination and minimizing or eliminating potential foodborne pathogens.

The frequent presence of insects and rodents in certain food vending areas is closely associated with contamination by yeasts and molds, adding an additional risk to the microbiological quality of food. This correlation is supported by other studies, which also highlight the hazards posed by these pests in food retail environments. For example, Al Mamun et al. (2013) observed contamination of sugary drinks sold roadside by *E. coli* , *Salmonella*, and *Shigella*, with sources of contamination including insects, dust, and water. Similarly Eromo et al. (2016) found contamination of roasted fish by *E. coli* , *Salmonella typhi* , *Pseudomonas* spp., and *Staphylococcus aureus* , largely due to the use of old newspapers for packaging, as well as the presence of insects and dust. These studies demonstrate the significant role insects can play in the transmission of bacteria to street foods, thereby facilitating cross‐contamination and increasing public health risks.

## 5 Conclusions

The results of this study highlight a concerning situation regarding hygiene practices in street food establishments in Marrakech. A significant proportion of vendors (72%) handle food with bare hands, and a substantial percentage of establishments (39%) do not meet the minimum sanitary standards for their operating environment. These practices, coupled with inadequate equipment, expose food to contamination risks, as evidenced by high levels of fecal coliforms (40%) and *E. coli* (28%) found in microbiological samples. While the absence of *Listeria* and the low presence of *Salmonella* are reassuring, the contamination by *E. coli* and *fecal coliforms* remains concerning, suggesting deficiencies in hand and utensil hygiene practices.

The findings also emphasize the impact of the vending environment on the microbiological quality of food. Less hygienic environments are directly associated with higher contamination rates by pathogens. Additionally, the advanced preparation of food, practiced by 70% of vendors, increases the risk of recontamination, underscoring the importance of maintaining hygiene throughout the entire food preparation and service chain.

To improve hygiene practices and reduce sanitary risks, several actions should be implemented: (a) Strengthening regulations and adopting an integrated approach to managing inspection activities in food establishments, (b) implementing training and awareness programs for street food vendors on hygiene principles, (c) improving sanitary and vending infrastructure, (d) ensuring the proper application of HACCP program guidelines, (e) mandating handwashing after any activity that may cause contamination, (f) replacing manual faucets and doors with automatic or easily operable models in cooking areas, (g) serving food immediately after cooking, (h) ensuring that cooked food is stored in appropriate containers protected from contamination. Additionally, efforts must be made to increase awareness of the importance of hygienic practices and food safety. It is also essential to regularly update and evaluate written protocols and procedures to effectively address health risks related to foodborne diseases.

## Author Contributions

**Morad Kaddouri:** conceptualization (equal), data curation (equal), investigation (equal), methodology (equal), software (equal), writing – original draft (equal). **Omar Ait El Alia:** validation (equal), writing – original draft (equal), writing – review and editing (equal). **Yassine Zine‐eddine:** data curation (equal), software (equal). **Ali S. Alqahtani:** formal analysis (equal), funding acquisition (equal), resources (equal), supervision (equal), validation (equal), visualization (equal), writing – review and editing (equal). **Mohamed Bouhrim:** validation (equal), writing – review and editing (equal). **Aziz Galman:** data curation (equal), software (equal). **Khalid Boutoial:** conceptualization (equal), formal analysis (equal), investigation (equal), supervision (equal), validation (equal), visualization (equal), writing – review and editing (equal).

## Conflicts of Interest

The authors declare no conflicts of interest.

## Data Availability

All data generated or analyzed in this study are available in the published article. For additional questions, contact the corresponding author.
